# Health Seeking Behavior Among Adults and Elderly With Chronic Health Condition(s) in Albania

**DOI:** 10.3389/fpubh.2021.616014

**Published:** 2021-03-16

**Authors:** Jonila Gabrani, Christian Schindler, Kaspar Wyss

**Affiliations:** ^1^Swiss Tropical and Public Health Institute, Basel, Switzerland; ^2^Faculty of Medicine, University of Basel, Basel, Switzerland

**Keywords:** non-communicable diseases, care-seeking behavior, elderly, primary healthcare, hospital

## Abstract

**Aim:** Assess the use of different health care service providers by adults (aged 18–59) and elderly (aged > =60) who suffer from non-communicable disease (NCD) and explore relationships between sociodemographic variables and care-seeking behaviors.

**Methods:** A cross-sectional survey was conducted in the districts of Diber and Fier in December 2018, using random cluster sampling. Descriptive statistics were used to compare the care-seeking behaviors of adults and elderly people. We employed binary and multinomial logistic regression to assess factors associated with the type of health service provider used. Analyses were adjusted for clustering within districts of residence.

**Results:** Out of 3,799 respondents, 1,116 (29.4%) suffered from an NCD. Of these, 95% sought to obtain care for their chronic condition through public healthcare providers. The elderly were more likely to use primary healthcare services (PHC) to initiate care when facing health problems (56%), compared to those aged 18–59 years (49%, *p* < 0,001). Over the last 8 weeks, 82% (914/1,116) of participants sought care. Binary and multinomial logistic regression analyses, adjusted for socio-demographic variables, showed that the elderly were more likely to choose PHC services (OR 1.56; 95% CI: 1.04; 2.35). Moreover, individuals who suffered from hypertension used PHC services more frequently than hospitals (OR 1.94; 95% CI: 1.32; 2.85). A positive association was found between living in an urban area and seeking care for NCDs at polyclinics (OR 10.1; 95% CI: 2.1; 50.1). There was no significant gender difference observed with regard to the type of provider consulted.

**Conclusion:** Public facilities were reported as the main providers for initiating care and the main providers used in the 8 weeks prior to the interview. While a majority of elderly people visited a PHC to initiate treatment (and follow up) on their chronic conditions, a substantial proportion of adults (aged 18–59) initiated and sought regular NCD care at a hospital. Educating patients and caregivers on active participation in NCD prevention, management, and control through the PHC level should be a long-term effort, along with the establishment of well-structured referral mechanisms and integrated care systems.

## Introduction

Non-communicable diseases (NCD) are an undisputed global challenge, increasing and multiplying with age, and are associated with higher and disproportional use patterns of health services (1–6). Care-seeking behaviors (e.g., initiating care at the right time, with the right provider; maintaining regularity of care seeking) are a prelude to a successful management of such conditions (7) These behaviors are influenced by a variety of factors: socioeconomic conditions, age, gender, financial means, their own perceived health status and illness, type of illness, as well as the available health services and access to them (7–9) Elderly people are particularly vulnerable to variability in their care-seeking patterns, including both over and underutilization of healthcare services. The elderly are also seen as dependent on their families or head of households (10–12). Indeed, adults and elderly with chronic conditions may receive care from multiple providers, across multiple settings, yet this care is often poorly structured (13). Studies have shown that 75–85% of conditions can be dealt with by utilizing primary health care (PHC) providers, while a consultation with a specialist is only necessary 10–12% of the time. Furthermore, only 5% of cases require a referral to higher levels of care (14). In order to better manage NCDs, emphasis is put on public health prevention policies through PHC institutions (3, 15). PHCs are a hub for continuity of care because they are the “first point of contact” and help patients coordinate their care across the system (2, 4, 16).

Albania, a South-Eastern European (SEE) country, is amongst the majority of European countries that regard the NCD epidemic as its most important public health challenge. The burden of chronic diseases (e.g., hypertension, cardiovascular diseases, and diabetes) are a central point of concern. In the decades ahead, it is predicted that the burden of chronic disease in Albania could increase further due to (i) lifestyle, (ii) lack of awareness of the disease, (iii) lack of the culture of prevention, inherited from the past ex-communist system, and (iv) heavily relying in curative care (polyclinics and hospitals) rather than prevention of the disease (17–20). Albania is also beginning to experience the effects of population aging. Starting from 2011 and looking ahead to 2060, the median age (33 years) is expected to increase by an additional 17 years. Elderly people in Albania face multiple challenges: changes in lifestyle that lead to smaller families, rural to urban migration, and decline of remittances from their children (21, 22). While the majority of healthcare in Albania is provided by the state, the private sector's importance is growing, mainly in urban areas. Governmental service provision is organized along three tiers: primary, secondary and tertiary healthcare services (18).

Access to PHC public services is free; since 2017, no citizen has been charged at the entry point of care, irrespective of their insurance status. Meanwhile, access to publicly financed outpatient specialist care requires a referral by a PHC provider. Outpatient specialist visits, with a PHC referral, are free of charge for people covered by the Mandatory Health Insurance Fund (MHIF). People without a PHC referral pay out of pocket based on tariffs set by the Ministry of Health and Social Protection (MoHSP), with tariffs varying by service. Those who are diagnosed with a condition can then either access inpatient care following the referral system and get free visits, or go directly to a specialist and pay the tariffs. Co-payments are applied to outpatient prescribed medicines, medical products and some diagnostic tests (23).

PHC services provides a package of basic medical aid: emergency care; mental health care; health services for children, women of reproductive age, adults and elderly people; health promotion and education (24). In order to implement each PHC service package, health centers are supplied with medical equipment, such as nebulizers, nasal speculums, otoscopes, ophthalmoscopes, glucometers, neurological hammers, stethoscopes, thermometers, and EKGs, etc (25). The list of essential drugs and consumables includes water for injections, atropine sulfate, dextrose, mannitol, diazepam, epinephrine, vitamins, dexamethasone, amoxicillin, etc (25).

In rural areas, a typical health center is staffed by up to three primary care doctors and nursing staff. By contrast, urban areas have PHCs as well as larger polyclinics that provide outpatient specialist care. These polyclinics serve sometimes as a first point of contact. In the private sector, principally in bigger urban settings, outpatient clinics offer a full range of medical services, anywhere from diagnostics to providing more comprehensive treatment and support (18, 26–28). The private sector requires out of pocket payments or possession of private health insurance. However, access to some private inpatient care services, such as nephrology and cardiac procedures, are also provided for publicly insured people (24, 25, 29).

In the last decade, a comprehensive approach has been employed by the Albanian government in order to tackle the toll of NCDs. With their aim being to strengthen and expand the role of primary health care (19), the government has done the following: (i) implementation of the national programme of free check-ups offered for all citizens aged 35–75 years (independent of their insurance coverage or health condition) (19), (ii) removal of all fees for medical visits at the PHC level for all citizens, and (iii) expansion of the list of reimbursed medicines, especially for chronic health condition(s) (28, 30–32).

Although there has been a positive trend of visits to primary care facilities over the last decade (33), there are claims that, despite the above policy initiatives to boost PHC service utilization, hospital doors are under “pressure” (20). In order to legally book a consultation in a hospital, known as “bypassing” the referral system, one must pay a fee of 21.5 Euro to the tertiary care institution; otherwise, the referral system is very strict in applying the rules that define a patient's pathway into the system (18, 20, 34, 35). Despite the evidence that NCDs can be managed at the PHC level, a fair amount of patients choose to bypass PHC services and consult directly with hospitals (20, 36).

The dynamics and patterns of care-seeking behaviors among adults and elderly people with NCDs remains scarcely documented in SEE countries, including Albania. As a result, we chose to investigate the first point of service use of persons suffering from an NCD in Albania.

### Frameworks for Studying Care-Seeking Behavior

There are different features that shape the healthcare utilization behaviors of patients. In order to understand decision making in health service utilization, classical models rely on identifying drivers that influence the choice (37). Among them, Andersen's Behavioral Model of Health Services Use is most often used in the literature. This model includes the predisposing factors age, sex and social structure, as well as “enabling” (e.g., distance to healthcare), and “generating need” (e.g., symptoms and impaired functioning) (38). In this study, we use the concept of access (first elaborated upon by Penchansky and Thomas), which summarizes a set of dimensions, describing the fit between the patient and the healthcare system (39). The specific dimensions of the framework were further operationalized by Obrist et al. (40) and applied in the Albanian context (41), namely: access, availability, affordability, adequacy and acceptability of healthcare services.

### Added Value and Relevance (Beyond National Context)

There is consistent international commitment to reducing the burden of NCDs (2, 19, 42). Multiple declarations have been made regarding the importance of the role PHC services play in preventing and controlling NCDs, especially in low and middle-income countries (LMIC). Designating primary health care as an avenue for the management of NCDs offers long-term, proactive, patient-centered and community-based care (3, 4, 15, 16).

While the importance of PHC services in LMIC is generally acknowledged, effective access and use of PHC services, as well as what drives NCD patients in LMIC, is not well-documented or researched (8, 15, 43).

This is also true for SEE countries, whose healthcare systems are in transition after having been previously focused on curative rather than preventive measures, and on infectious rather than non-communicable diseases (9). Unlike many other studies in the western context, where both NCDs and PHC services are well-investigated, most healthcare systems in SEE countries have limited access to, and use of, quality data for informing policy (2, 42).

Against this background, this study provides new evidence for understanding the care-seeking behavior of adults and elderly people suffering from NCDs in LIMC countries. More explicitly, it focuses on the utilization of primary care vs. hospitals for initiating care and following up on the chronic conditions (9, 44).

The aims of this study are to assess the health seeking patterns of adults (aged 18–59) who suffer from NCDs and compare them to the patterns of elderly people (aged > =60) in order to establish a possible relationship between sociodemographic variables and care-seeking behaviors.

## Methods

### Study Design and Area

The data for this study were collected by the Household Survey within the “Health for All” (HAP) project in Albania, funded by the Swiss Agency for Development and Cooperation (45). The overall goal of the project is to help the Albanian population achieve better health through improved primary health care services and health promotion activities that are directed at prevention and treatment of NCDs.

The household cross-sectional survey was conducted in December 2018 in two regions: (1) Fier, a partially industrial region located South-West of the capital, Tirana, with access to the seaside and Diber, a relatively poor, mountainous region located in the eastern part of the country bordering North Macedonia. The regions of study are also described in two recent publications (41, 45, 46). The two regions make up around 16% of the territory of Albania and demographically represent around 15.7% of the population (447,263 out of 2.8 million). The 2011 census registered 310,277 people living in 87,605 households in Fier, and 137,036 people living in 33,204 households in Diber (45). Distribution of governmental services, such as PHC centers and regional hospitals, are comparable in both regions; however, in Diber there are no licensed private hospitals or clinics, and the geographical proximity/accessibility of health facilities is lower than in Fier. These regions are representative of Albania, with the exception of the capital city of Tirana.

Supplementary Image 1: Territorial administrative map of Albania.

### Study Population

The study population consisted of adults, aged 18 years and above who reported suffering from NCDs such as hypertension, heart problems (CVD), diabetes, rheumatism, respiratory diseases and diseases of the nervous system, mental health, stroke and cancer. Participants resided in selected households, and consented to take part in the study. Information on sociodemographic characteristics, type of illness, diagnosis and health seeking behavior were obtained.

### Sampling

This survey was based on a cross-sectional cluster sample design using population estimates from the 2011 census, extrapolated to 2018. Sampling was conducted in a two-stage approach to obtain representative data for the two regions of Albania that were covered (stratified by urban and rural areas).

The sample size calculation was based on estimating important prevalence with sufficient precision in each of the two districts. For instance, a true outcome prevalence of 10% was to be estimated within an error margin of 3%, with 95% certainty. Based on experience, we assumed a cluster size of 12 households and an intraclass correlation of 0.04 would provide a design effect of 1.44, rounded to 1.5. With an assumed non-participation rate of 10%, this resulted in 53 clusters, or 634 households, per district.

The required number of clusters was obtained according to the formula:

$$\begin{array}{l} k=1.1\times \left(1.96^{\wedge }2\times \text{p}\times \left(1-\text{p}\right)\right)/\left(\text{B}\times {\text{m}}^{\wedge }2\right)\times \text{DF}, \end{array}$$

where *k* = required number of clusters, *p* = 0.1 (true prevalence), B = 12 (cluster size), m = 0.03 (error margin) and DF = 1.5 (design factor).

Households were included if at least one person who resided there suffered from a chronic condition or disability that lasted more than 3 months. Within each household, the “head of household” was asked about characteristics of the home and its members, including health and insurance status. We then randomly selected one individual to obtain information regarding their type of illness, diagnosis and health seeking behavior.

### Questionnaire Development

The questionnaire was designed to provide relevant information on health patterns and care-seeking behaviors of adults and elderly people suffering from NCDs. The questionnaire applied was divided into two parts: (i) household questions, such as distances to health facilities and services at community level, household characteristics, etc. and (ii) individual questions for persons with at least one chronic condition, including socio demographic information (e.g., age, gender, education, employment, insurance and respective NCD(s), and specific information on disease patterns and health seeking behavior. The primary outcome was the type of provider that individuals with NCDs visited over the past 4–8 weeks.

Specifically, the questionnaire included items investigating three main areas of interest: (1) service provider where the patient initiated care, (2) the place of diagnosis of their NCDs, and (3) service provider consulted within the 8 weeks prior to the interview. Information about the patients' first contact was obtained by asking them which health facility they would most likely go to for initial medical assistance when facing health problems. Regarding the most recent visits, we asked patients to further specify the healthcare provider they consulted within the last 8 weeks (related to their NCD care needs). In the analysis, we only looked at the first service use and not on multiple service uses. Respondents with chronic conditions who did not exclusively use PHCs were asked about the reasons for bypassing PHCs.

### Data Collection

Data collection was carried out between the 7th and 20th of December 2018 by 16 interviewers, organized into four teams. Each team was headed by one supervisor who was responsible for the organization of the team and quality assurance of the data collection process. Before data collection, interviewers and supervisors were trained in a two-and-a-half-day course, which included a pretest. Data collection was done using electronic data capture with tablets, equipped with Open Data Kit software (ODK), and was handled through a structured questionnaire. Respondents were asked questions without hearing the survey choices (i.e., respondents were given the chance to respond freely). Based on the answers given by the respondents, respective categories were ticked by the interviewers. In other cases, the answer categories were prompted by the interviewers from the ODK form (47).

### Statistical Analysis

Data were analyzed using STATA, version 14 (Stata Corp, College Station, TX, USA). The respondents participating in the survey were stratified into two main groups, namely adults (aged 18–59) and elderly (aged > =60) who reported having an NCD. The care-seeking patterns were assessed through descriptive statistics and binary and multinomial logistic regression models. The dependent variable was the type of facility utilized (primary care settings vs. hospitals) for regular care seeking during the last 2 months. The health care providers included district level health care facilities.

Binary logistic regression models were used to identify factors associated with the preference for attending a primary care health facility as opposed to a public hospital. These analyses were then refined by distinguishing different types of primary care facilities (i.e., PHC facility, a polyclinic or health post) in multinomial logistic regression models. Based on existing literature and our research questions, we included both socio-economic and health characteristics of the respondents, such as (1) gender (male or female), (2) age group (18–59 or > =60 years), (3) residency (urban or rural), (4) health insurance and socioeconomic beneficiary status (yes or no), and (5) the respective type of NCD. Region of study was further included to adjust for unobserved confounders differing between the two regions. Moreover, we investigated the relationship between different reported conditions and the use of PHC settings as regular NCD providers. Standard errors of parameter estimates were adjusted for potential clustering of the data within communities of residence. Results of the multinomial logistic regression models are expressed as relative risk ratios (RRR) which can also be interpreted as odds ratios (ORs) between the specific outcome level and the reference outcome level for the respective factors. In our analyses, we always used “public hospital” as a reference level.

### Ethical Considerations

The study protocol and questionnaires received ethical clearance from the MoHSP on the 8th of October 2018, Nr. prot.5800.

Given the lack of preference of the patients for written consent, mainly due to their reluctance to share personal information, oral informed consent was obtained from all respondents at the beginning of the interview. It was pointed out that participation was voluntary and that the respondents could withdraw their participation at any time. The head of household was also provided with an informational letter on the objective and the purpose of the survey and aspects relating to the confidentiality of information.

## Results

### General Characteristics of the Households

Overall, we established contacts with 1,371 eligible households, 82 (6%) of which did not consent to participate. This resulted in 1,289 households. From 3,799 adult individuals living in these households, 1,116 (29. 4%) suffered from an NCD.

Nearly two-thirds (64%) of the households were located in rural areas. The average number of household members was 3.4 persons (SD 1.8). The most common sources of income for households were governmental pensions (59%), followed by farming/livestock (33%), remittances (27%), salary (26%), social aid (20%), and private business (13%). Average age of household members was 41 years. More than half of the individuals were married (58%) and more than one third were single (34%). The most common educational degree obtained was completion of secondary school (grades 6–9, 43%), while others had a high school degree (grades 10–12, 30%).

### Socio-Demographic Characteristics of the NCD Respondents by Age Category

Table 1 presents the characteristics of the respondents who provided information on their chronic condition(s). Of the 1,116 interviewees, 63% were females, 41% were between the ages of 18–59, and another 59% were more than 60 years old.

**Table 1:** Patients' socio-demographic characteristics by age category.

**Characteristics**	**Total population (*n* = 1,116)**		**Aged 18–59 Yyears (*n* = 447)**		**Aged> = 60 years (*n* = 669)**	
	***n***	**%**	***n***	**%**	***n***	**%**
**Gender**						
Male	407	36	144	33	263	39
Female	709	64	303	67	406	61
**Age**						
18–59 years	447	40	447	40		
≥60 years	669	60			669	60
**Education**						
None	12	1	3	1	9	1
pre-school/kindergarten	2	0	0	0	2	0
primary (grade 1–5)	193	19	10	2	183	30
secondary grade (grade 6–9)	495	48	238	55	257	42
high school	268	26	142	33	126	21
Technical/college	13	1	5	1	8	1
University	57	5	31	7	26	4
**Source of income**						
Private business in Albania	144	7	81	11	63	5
Salary	266	13	140	18	126	10
Pension	733	35	134	17	599	46
Social aid	209	10	111	14	98	8
Farming/livestock	371	18	181	24	190	15
Remittances	307	15	101	13	206	16
Other	38	2	22	3	16	1
**Marital Status**						
Married	832	75	391	87	441	66
Divorced	7	1	6	1	1	0
Separated	1	0	0	0	1	0
widow/er	241	22	20	4	221	33
Single	35	3	30	7	5	1
**Health Insurance**						
Yes	958	86	339	76	619	93
No	158	14	108	24	50	7
**Benefiting soc. Aid**						
Yes	229	21	124	28	105	16
No	887	79	323	72	564	84
**Location of Residence**						
Rural	712	64	282	63	430	64
Urban	404	36	165	37	239	36

In both age groups, most people lived in rural areas (64%). The age distribution was similar in urban and rural areas. Older participants were more likely to have a low education level (primary education or below) than younger participants. The main sources of income were pensions and remittances for the older age group (elderly) and farming or salaried activities for the younger age group (adults). The coverage of health insurance was higher among the elderly.

### Self-Reported NCDs Among Adults and Elderly People

**Figure 1:**
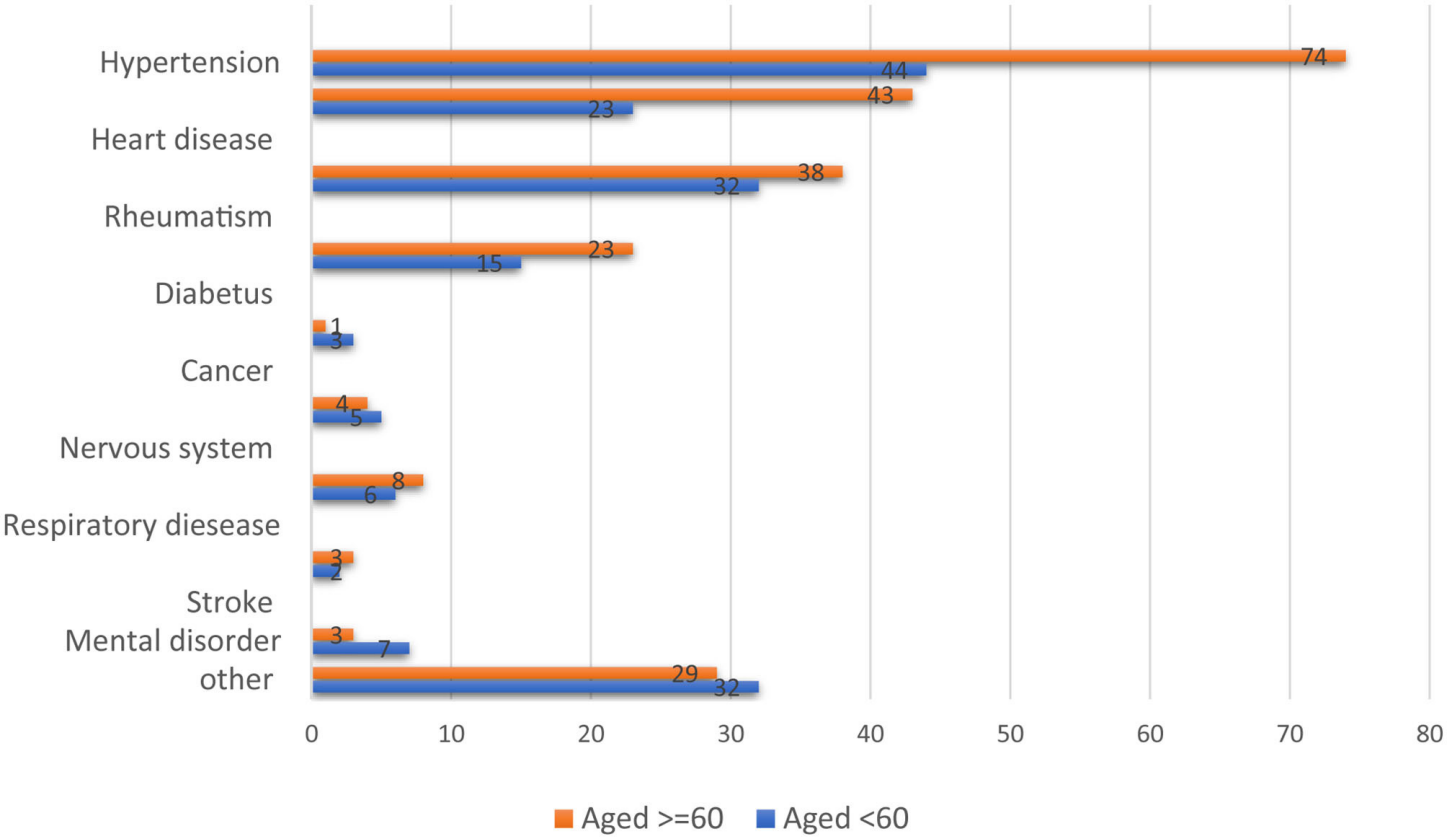
Frequency of self-reported NCDs among adults and elderly (numbers in percentages).

Figure 1 presents the self-reported NCDs among the study respondents. Prevailing chronic conditions among adults and elderly were: high blood pressure (adults 44%, elderly 74%), heart problems (adults 23%, elderly 43%), rheumatism (adults 32%, elderly 38%), and diabetes (adults 15%, elderly 23%). Chronic conditions were more common among females than males for rheumatism (women 44%, men 22%) and high blood pressure (women 69%, men 50%); no substantial gender differences were identified for the other various chronic conditions.

About one-third of the interviewees (344/1,116) reported having two chronic conditions, 35% (235/669) of whom were the elderly group and 24% (109/447) the adult group; from there, 16% (180/1,116) indicated having three conditions and 5% (60/1,116) indicated having four or more chronic conditions. The respective percentages were consistently higher in the elderly group than in the adult group. Prevailing paired combinations were hypertension and heart problem (31%), hypertension and rheumatism (31%), hypertension and diabetes (15%), and heart problems and rheumatism (5%).

### Initiating Care

Of our interviewees, 96% of them used public sector health providers for initiating care. Only 4% declared that they received care through private providers, typically by a private hospital or clinic (75%).

Table 2 shows the patterns of health facility utilization, categorized by age, in a broad spectrum of care seeking. Overall, 547 people (53% of the 1,078 total sample who sought care at the public health sector) responded that they initially sought care at a health center when facing health problems, followed by hospitals (33%), health posts (12%), and polyclinics (2%).

**Table 2:** Types of clinics attended by adults and elderly with NCD(s) for initiating care, establishing diagnoses, and seeking regular care (follow up)^*^.

	**Total population**		**Aged 18–59 years**		**Aged> = 60 years**		P*
**Total *n*= 1,078**	**Obs. Nr**.	**%**	**Obs. Nr**.	**%**	**Obs. Nr**.	**%**	
**Initiate care**	*1078*		*N = 427*		*n = 651*		* < 0.001*
Governmental hospital	351	33	171	40	180	28	
Governmental policlinic	23	2	5	1	18	3	
Governmental health center	574	53	208	49	366	56	
Health Post	130	12	43	10	87	13	
**Establish diagnose**							
*Total n= 1,058*			*n=414*		*n=644*		* < 0.001*
Governmental hospital	678	64	278	67	400	62	
Governmental policlinic	47	4	13	3	34	5	
Governmental health center	298	28	113	27	185	29	
Health Post	35	3	10	2	25	4	
**NCD care over 8 weeks**							
*total n= 790*			*n= 294*		*n=496*		* < 0.001*
Governmental hospital	295	37	135	46	160	32	
Governmental policlinic	37	5	15	5	22	4	
Governmental health center	416	53	127	43	289	58	
Health Post	42	5	17	6	25	5	

*p-values using Chi square tests*.

The proportion of persons using a hospital for initiating care was 40% among adults and 28% among the elderly (*p* < 0.01, see Table 2). Conversely, the elderly were more likely to attend governmental PHC health centers and affiliated health posts for initiating care (elderly 56% vs. adults 49%, *p* < 0.001).

The study participants established their laboratory diagnosis mainly through hospitals (64%), followed by PHC centers (28%) and polyclinics (4%); there was little variation between the two age groups in terms of proportions.

### Use of Health Care Within the Last 8 Weeks

Out of 1,116 adult participants with a chronic condition, 82% of respondents consulted a health service provider over the past 2 months. Contrastingly, 18% did not seek care or relied on self-treatment.

Of 914 people who sought care over the last 8 weeks, 790 people indicated having consulted a public provider (see Table 2). Among them, respectively, 53% consulted PHCs, 37% consulted hospitals, 5% consulted health posts, and 5% consulted polyclinics. Those in the adult group were more likely to frequent a hospital (adults 46% vs. elderly 32%, *p* < 0.01). Oppositely, those in the elderly group were more likely to attend governmental PHC health centers and affiliated health posts during the eight weeks preceding the interview (elderly 58% vs. adults 43%, *p* < 0.001). Patients were most commonly treated by a doctor and/or a nurse.

Participants had certain reasons for choosing their respective providers for NCD treatment, those of which include geographical proximity to the health provider (49%), low costs (37%), good services (27%), insurance coverage (24%), familiarity with the staff (22%), quality of care (19%), and well-qualified staff (15%).

### Frequency of Health Seeking Behavior

Out of 1,116 persons with a chronic condition, 44% (*n* = 495) sought care once per month. Another 35% (*n* = 391) sought care several times per year. In general, the health services utilization was higher among the elderly, who predominantly sought care once per month [52% (*n* = 349)]. Compare this to the adults, who mostly sought care several times per year for their chronic condition [46% (*n* = 207)] (Table 3).

**Table 3:** Frequency and regularity of health seeking behavior for chronic condition among adults and elderly (*n* = 1,116).

	**Total pop**.		**Aged 18–59 years**		**Aged> = 60 years**	
	**Freq**.	**Percent**	**Freq**.	**Percent**	**Freq**.	**Percent**
Does not seek care	34	3	19	4	15	2
Several times per month	117	10	43	10	74	11
Once per month	495	44	146	33	349	52
Several times per year	394	35	207	46	187	28
Once per year	57	5	27	6	30	4
Less regular than once per year	19	2	5	1	14	2
Total	1,116	100	447	100	669	100

The main reasons for not seeking care were related to self-medication and the belief that the health problem would go away without medical treatment. Lack of financial funds was another aspect that patients mentioned, as well as the lack of time/transport.

### Factors Associated With the Use of Health Service Provider Within the Last 8 Weeks

Given that the vast majority of the study participants reported utilizing governmental providers, the present study solely focuses on those who have consulted a governmental service and further assesses the level of care and possible association with their sociodemographic characteristics. In order to establish such an association, logistic and multivariate regression methods were employed.

Because the respondents could indicate multiple levels of services used, we set a condition in the regression model to include those who mentioned one choice. This resulted in a total of 698 observations included in the analysis.

Table 4 shows the observed associations of socioeconomic factors (e.g., age, household socioeconomic beneficiary status, location of residence, type of chronic condition, health insurance, marital status) with the type of healthcare facility chosen for the provision of the NCD follow up.

**Table 4:** Odds ratios of using primary care facilities as opposed to public hospitals for NCD-care in the preceding 8 weeks associated with different personal characteristics.

**Variables /Factors**		**+Any primary care setting (a, b or c)**		**Policlinic (a)**		**PHC (b)**		**Health Posts (c)**		^**^p	^***^p
		**OR**	**95% CI**	**OR**	**95% CI**	**OR**	**95% CI**	**OR**	**95% CI**		
**Gender**	Male										
	Female	0.97	0.66; 1.42	1.40	0.29;6.63	0.99	0.67; 1.49	0.54	0.21; 1.34		
**Age**	<60 year										
	> =60 year	1.56	1.04; 2.35	0.67	0.10; 4.15	1.67	1.10; 2.53	0.76	0.29; 2.00	0.03	0.01^b^
**Marital Status**	Married										
	Widower	0.97	0.62; 1.51	3.85	0.84; 17.6	0.89	0.57; 1.40	1.81	0.62; 5.25		0.08^a^ 0.02^c^
	Single/divorced	1.09	0.40; 2.99	0.74	0.02; 23.7	1.38	0.49; 3.87	0	0		
**H. Insurance**	Yes										
	No	1.08	0.51; 1.87	0	0	1	0.56; 1.76	3.26	1.15; 9.27		0.03^c^
**Socio,econ.Aid**	No										
	Yes	0.79	0.51; 1.21	1.42	0.25; 7.80	0.73	0.47; 1.14	1.29	0.45; 3.63		0.03^b^
**Location**	Rural										
	Urban	0.93	0.65; 1.33	10.1	2.1; 50.1	0.91	0.63; 1.31	0.42	0.14; 1.21		0.05^a^
**Ch.Conditions**											
Hypertension	y vs. n	1.94	1.32; 2.85	0.60	0.15; 2.48	1.94	1.31; 2.88	3.31	1.20; 9.01	0.01	0.08^b^
Diabetes	y vs. n	0.71	0.46; 1.08	0.29	0.05; 1.61	0.73	0.47; 1.12	0.61	0.19; 1.91		
Heart problems	y vs. n	0.69	0.48; 0.98	0.24	0.05; 1.04	0.74	0.51; 1.05	0.41	0.16; 1.03		
Stroke	y vs. n	0.55	0.17; 1.69	0	0	0.65	0.21; 2.01	0	0		
Cancer	y vs. n	0.84	0.24; 2.90	0	0	1.01	0.29; 3.45	0	0		
Respiratory diseases	y vs. n	1.54	0.78; 3.03	0.66	0.06; 6.92	1.50	0.75; 2.98	2.33	0.57; 9.47		
Mental disorder	y vs. n	1.67	0.59; 4.73	1.51	0.05 43.4	1.25	0.41; 3.81	9.57	1.75; 52		0.01^c^
Nervous system	y vs. n	1.20	0.50; 2.87	1.72	0.18; 16.7	1.29	0.53; 3.12	0	0		
Rheumatism	y vs. n	0.86	0.60; 1.23	1.66	0.48; 5.76	0.85	0.59; 1.22	0.69	0.29; 1.63		

* *RRR (relative risk ratio) ratio between the relative risk of attending the respective facility between patients with and without the respective socioeconomic characteristic or disease and the corresponding relative risk of attending a public hospital*.

** *p-value of likelihood-ratio test of the association of the respective factor with the odds of choosing any primary care setting as opposed to a public hospital (results from the Logistic regression)*.

*** *p-value of likelihood-ratio test of the association of the respective factor with the odds of choosing a specific primary care setting (a, b, or c) as opposed to a public hospital (results from the Multinomial logistic regression)*.

+ *Primary care, Any Primary care setting (of a, b, or c)*.

After adjusting for the sociodemographic factors, the elderly group were consistently more likely to choose a primary healthcare setting. Given the choice between attending a primary health care facility or a public hospital, the odds that elderly people opt for a primary care setting increase by a factor of 1.56 (95% CI:1.04; 2.35) and by a factor of 1.67 (95% CI: 1.10; 2.53) for PHCs, compared to the choice of attending a hospital for NCD follow-up care.

Regarding the role of marital status, widows were more likely than married people to follow up on their chronic health conditions at polyclinics (OR 3.85; 95% CI: 0.84; 17.6). Additionally, a positive association was found between living in an urban residence and seeking regular NCD care at polyclinics (OR 10.1; 95% CI: 2.1; 50.1).

Individuals who suffered from hypertension tended to regularly follow up on their condition at the primary care level, as opposed to a public hospital, especially at a PHC (OR 1.94; 95% CI: 1.31; 2.88) or health post (OR 3.31; 95% CI: 1.20; 9.01).

A positive association was observed between people with no health insurance and the preference for health posts for managing chronic health conditions (OR 3.26; 95% CI: 1.15; 9.27). There was no significant gender difference observed with regard to opting for a primary health care setting (polyclinics, PHC or Health posts).

### Reasons for Use of PHC Services and Other Providers Simultaneously

From the total sample, 985 respondents (87%) reported having a facility closer to the (than the nearest hospital (1–5 km). Patients often chose to visit both PHC facilities and public hospitals because they had been referred by their doctor (adults 31%; elderly 26%) or that tests (adults 26%; elderly 22%) or services had not been offered at the level of the PHC provider (adults 16%; elderly 16%). Almost 7% of patients with a chronic condition chose to exclusively (or occasionally) visit a public hospital, due to their perception of a doctor's competence and perceived poor quality of services (see Table 5).

**Table 5:** Reasons for not using PHC services.

Reasons for non-utilization of PHC (*N* = 1205)^*^	**Aged 18–59 years**		**Aged> = 60 years**	
	***n***	**%**	***n***	**%**
Services are not offered	89	16	109	16
Too far, no transport	3	1	20	3
Not competent staff	34	6	43	6
Not all tests could be conducted	141	26	145	22
I was referred (to specialist doctor)	167	31	173	26
I know the other doctor	21	4	44	7
Facility closed	22	4	29	4
Poor service	47	9	50	8
Other	19	3	49	8
Total population	543	45	662	55

* *(N=1,205) respondents have chosen more than one answer*.

## Discussion

This study adds evidence to the care-seeking patterns and health service consultations among adults and elderly people suffering from NCDs in LMICs, responding to the recent call for more empirical research to understand the health service utilization by patients with NCDs in LIMCs (43).

Several studies have investigated health services utilization patterns of people with NCDs at PHC and hospital level in near-by countries (48–50). as well as in more distant countries with different systems (51). The questionnaire used for this study was previously used in the frame of study in 2015 in Albania (45). The questionnaire and study variables are comparable to the previous similar studies such as those conducted in Serbia, Bosnia and Hercegovina, and Greece (48–50).

Our findings provide new evidence on drivers of the use of PHC services by patients with chronic conditions, and thus contributes to the international debate for moving toward Universal Health Coverage (2, 17, 22, 42, 43).

### Initiating Care

The provider of choice for initiating care for patients with NCDs were either a government PHC (e.g., health centers, polyclinics, and health post) or a public hospital. The reasons for these choices were: geographical proximity, low costs, health insurance coverage, and quality of services. These findings are similar to those from previous studies which showed that such facilities are the first point of care for patients with any disease in Albania (28, 45).

The preference for PHC institutions when initiating care has also been found in studies undertaken in other settings where primary care is either evolving, or is a vital pillar of the overall health system. However, the findings are not conclusive; there is concern that primary care facilities remain underutilized in settings where the PHC is less consolidated (48–51).

The behavior among adults and the elderly population related to “the initial point of care” varied; a greater proportion of adults (40%) suffering from NCDs initiated care directly at the hospital level through self-referral, bypassing PHC services. The study results indicate that adults frequently go directly to the hospital, albeit associated with higher costs. This finding has also been stated in previous research conducted (20). There is evidence that older and less educated patients are more likely to follow the advice of their PHC provider, and are therefore less likely to bypass the gatekeeping system (52, 53).

Higher-level public hospitals in Albania are largely perceived to have better health resources, both in terms of workforce and diagnostics, and a greater ability to provide quality of services compared to public health facilities at lower-levels. This could explain why a relatively high proportion of adults decide to initiate care directly at the hospital level (20).

### Use of Health Care Providers Within the Previous 8 Weeks

The study results indicate that, given the choice between attending primary care facilities or a public hospital, the odds that elderly people would use the primary care facilities were consistently higher compared to adults. Patients who chose to consult with other providers over consulting with PHC facilities (e.g., polyclinics, specialists in hospitals) did so mainly because they were either referred, not all tests were available, or services were not offered.

These findings indicate an opportunity to provide specific NCD screening and management programs in primary healthcare facilities. NCD screening and diagnostic services could be similarly provided through existing government healthcare facilities (i.e., primary healthcare centers and health posts). Thus, having well-established referral patterns and integrated service models where both specialists at polyclinics or hospitals, and also family doctors at PHCs, hold a role and are related through well-structured systems to each other, would increase the effectiveness and efficiency to manage chronic conditions. Updated protocols, along with mechanisms ensuring their effective use (e.g., electronic decision support systems), may tackle both the high referral rate and the bypassing of the PHC system, thereby increasing the potential for primary health care to better contribute to NCD follow-up.

Therefore, continuous professional development systems should ensure that the knowledge and skills of healthcare professionals are regularly updated, and that essential NCD services, including those relating to the elderly and mental health conditions, are provided with good quality.

No association was found between insurance status and primary healthcare service utilization. In fact, since 2016, even the uninsured population suffering from an NCD are entitled to drug reimbursement schemes. All the patients are eligible to have free medical care through a family physician (i.e., PHC) if they have a personal ID. They are also eligible for an initial diagnosis from a specialist, (if assumed to be chronic disease patients) after which they follow the referral system from PHC and onwards. Moreover, first choice treatments (i.e., drugs), which are ordered by the specialist and prescribed by the family doctor at a PHC facility, are reimbursed. Consequently, a health insurance card is mainly necessary for obtaining additional tests and treatment options at the level of PHC and/or specialty services.

We found that 18% of people who were chronically ill did not consult a service within the previous 8 weeks of the interview, thus exhibiting a lack of a regular care-seeking behavior or relying in self-treated, for example by going directly to a pharmacist.

## Limitations of the Study

### Health Seeking Behaviors and the Respective NCDs Relied Entirely Upon Self-Reporting

Given the age distribution of the patients suffering from NCDs, a higher number of elderly people are present in the sample (which is expected, although it might imply some underrepresentation of younger adults). The differentiation between high blood pressure and heart problems may have led to some confusion in the way lay persons use concepts of circulatory problems (high blood pressure) and cardiac/heart problems (ischemia, for instance). The regions where the study was conducted represent both the mountainous and coastal regions of Albania. Given the socio-cultural and economic diversity of the country, care-seeking patterns in major urban cities, namely Tirana, are likely to differ. Complementary qualitative research may be conducted in the future, including in-depth interviews or focus group discussions. This could aid in the investigation of health seeking pathways and identify reasons why chronically ill patients choose to by-pass PHC services and consult directly at hospital level.

## Conclusions

This study indicates that, in the two regions of study, ~90% of the households' healthcare demands for NCD management was addressed by the public health sector, often via the primary care level and public hospitals.

Patients most commonly chose to visit both PHC facilities and hospitals because they were referred, or because of the lack of tests/services accessible to them at the PHC level of care. While elderly people most frequently initiated treatment and followed up on their respective chronic conditions at the PHC level, a substantial number of adults initiated and sought regular NCD care at the hospital level. This would indicate a propensity for younger NCD patients to choose more facilities with higher-level healthcare than is actually necessary, compared to the elderly group.

Primary health care services were more likely to be the regular NCD care provider for people suffering from hypertension. Meanwhile, polyclinics were more likely to be used among those who suffered from conditions such as mental disorder, stroke and cancer. Participants living in urban areas were more likely to seek regular care at polyclinics compared to their counterparts living in rural areas.

In order to foster and scale management of chronically ill patients in primary care settings in Albania, there is a need for (i) updated protocols on standardized procedures of NCD treatment for adults/elderly people and systems assuring their effective use, (ii) increased referral support, (iii) essential diagnostic tools, (iv) skillful health workforce at PHC level who are able to manage and coordinate NCD care, and (v) raising of population awareness on the benefits of primary care for the integrated management of chronic conditions.

## Consent for Publication

Oral informed consent was obtained from all respondents.

## Data Availability Statement

Data are available on reasonable request and approval by the authors and involved project partners.

## Ethics Statement

The study protocol was approved by the ethics committee of north-western and central Switzerland (EKNZ- Ethikkommission Nordwest- und Zentralschweiz), No. 30 715, and the study protocol and questionnaires received ethical clearance from the MoHSP on the 8th of October 2018, Nr. prot.5800. Given the lack of preference of the patients for written consent, mainly due to their reluctance to share personal information, oral informed consent was obtained from all respondents at the beginning of the interview. It was pointed out that participation was voluntary and that the respondents could withdraw their participation at any time. The head of the household was also provided with an informational letter on the objective and the purpose of the survey and aspects relating to the confidentiality of information.

## Author Contributions

JG and KW: conceptualization. JG and CS: data curation, formal analysis, and methodology. JG: writing—original draft. CS and KW: Review and editing. KW made substantial contribution on critically revising the manuscript for important intellectual content. All authors contributed substantially to manuscript and agreed to the final version.

## Conflict of Interest

The authors declare that the research was conducted in the absence of any commercial or financial relationships that could be construed as a potential conflict of interest.

## Abbreviations

PHC: Primary healthcare
UHC: Universal Health Coverage
NCDs: Non-communicable diseases
ADHS: Demographic and Health Surveys
MoHSP: Ministry of Health and Social Protection
MHIF: Mandatory Health Insurance Fund
WHO: World Health Organization
ODK: Open Data Kit
HC: Health centers
LIMC: Low- and Middle- Income Countries
EU: European Union
SEE: South-Eastern European countries.
