# Supplementary figures and images for: Health Seeking Behavior Among Adults and Elderly With Chronic Health Condition(s) in Albania
Source: Front Public Health. 2021 Mar 16;9:616014. doi: 10.3389/fpubh.2021.616014 (PMC8007873; doi:10.3389/fpubh.2021.616014)

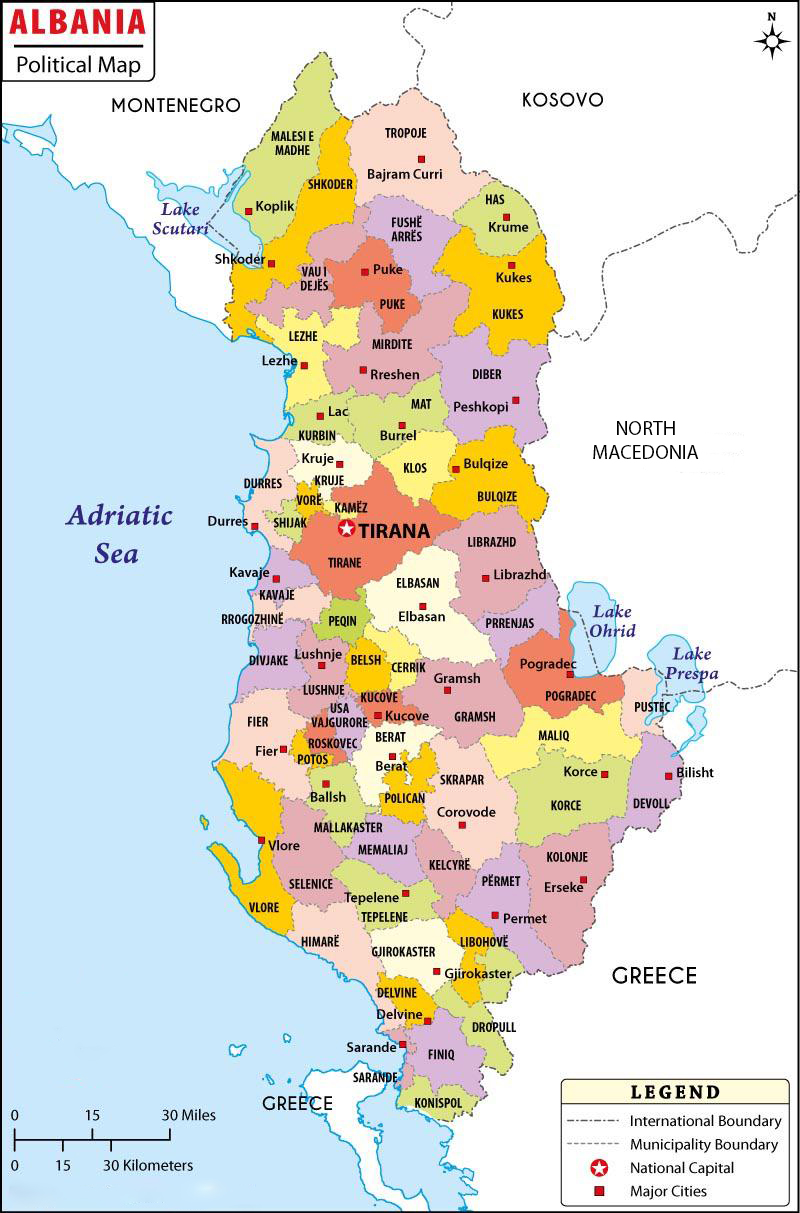

Supplement: Supplementary file 1 [file Image_1.JPEG]
